# Differential Influence of Acupuncture Somatosensory and Cognitive/Affective Components on Functional Brain Connectivity and Pain Reduction During Low Back Pain State

**DOI:** 10.3389/fnins.2019.01062

**Published:** 2019-10-04

**Authors:** Jeungchan Lee, Seulgi Eun, Jieun Kim, Jun-Hwan Lee, Kyungmo Park

**Affiliations:** ^1^Athinoula A. Martinos Center for Biomedical Imaging, Department of Radiology, Massachusetts General Hospital, Boston, MA, United States; ^2^Department of Biomedical Engineering, Kyung Hee University, Yongin, South Korea; ^3^Clinical Research Division, Korea Institute of Oriental Medicine, Daejeon, South Korea

**Keywords:** functional connectivity, default mode network, sensorimotor network, salience network, somatosensory afference, needling credibility

## Abstract

The underlying mechanism of pain reduction by acupuncture is still unclear, because acupuncture treatment involves multidimensional factors. In this study, we investigated the differential influence of acupuncture components on brain functional connectivity and on pain reduction. We used a specific form of sham acupuncture (phantom acupuncture; PHNT), which only has a needling-credibility (a belief that they were treated with real acupuncture needles), while real acupuncture (REAL) has a somatosensory needling stimulation, as well as a needling-credibility. Forty-three patients with low back pain were randomized into the REAL group (*n* = 25) and the PHNT group (*n* = 18). They underwent two pain steady-state fMRI runs implemented by a low back extension (LBE) pain model (lifting the low back using air-cuff inflation) before and after REAL or PHNT stimulation. Subjective pain ratings, perceived throughout the LBE runs due to the posture, were reported (LBEpain). The regions of interest (ROI) were (1) the main nodes of the default mode network (DMN) – the medial prefrontal cortex (mPFC), posterior cingulate cortex (PCC), (2) the main nodes of the salience network (SN) – the anterior/posterior insular cortices (a/pINS), and (3) the low back-specific region of sensorimotor network (SMN), S1_back_. Significant reductions in LBEpain were observed in both groups (REAL = −1.02 ± 1.53, PHNT = −1.26 ± 2.20). In REAL group, decreased LBEpain was positively correlated with decreased functional connectivity between the mPFC and pINS (*r* = 0.58, *P* < 0.05). Reduced LBEpain in PHNT was negatively correlated with increased PCC–aINS connectivity (*r* = −0.48, *P* < 0.05) and tended toward positive correlation with decreased S1_back_–pINS connectivity (*r* = 0.44, *P* = 0.07). Our findings might suggest different brain mechanisms of observed pain reduction; REAL seems to involve detachment of the self from the sensory aspect of pain, while PHNT does to shift attention to self and disengages physical pain processing hubs. This exploratory study proposes a sham methodology to dissociate the influence of different acupuncture components in acupuncture research. Further studies need to be followed with more elaborated hypothesis, study design, and analysis considering various cognitive/affective factors for better understanding of brain mechanisms of pain reduction regarding the different acupuncture aspects.

## Introduction

Acupuncture treatment, sham as well as real, is known to modulate pain, but the underlying brain mechanism is not clear, probably because multidimensional factors are involved (Kaptchuk, 2011), and it is not easy to dissociated them from each other. To provide a standard for investigation into the acupuncture mechanism, acupuncture components have been defined as needling-specific (e.g., somatosensory needling, which is exclusive to acupuncture needling such as insertion and manipulation), specific non-needling (e.g., theory-based diagnosis and palpation, which has been considered to be related to treatment efficacy), and non-specific [e.g., needling credibility (patients’ belief that they were treated with real acupuncture needles) and visual feedback (observation of treatment procedure), which is not exclusively driven by or related to needling itself] components (Langevin et al., 2011). However, it has not yet been possible to differentiate needling-specific from non-specific components, because tactile (touch) stimulation cannot be excluded as a factor in the proposed sham acupunctures (Lee et al., 2014; Makary et al., 2018). Thus, to differentiate acupuncture needling-specific (somatosensory needling) from non-specific components (needling credibility and visual feedback) (Langevin et al., 2011), phantom acupuncture (PHNT), which can produce non-specific effects without the acupuncture-specific components, has been devised to contrast with real acupuncture (REAL) (Lee et al., 2014; Makary et al., 2018). The comparison between real and PHNT stimulation has shown that somatosensory needling stimulation (acupuncture needling-specific components) produces sympathetic activation (e.g., greater skin conductance response) as well as greater acupuncture-related sensations (overall acupuncture sensation measured as Mass Index (Kong et al., 2007), soreness, tingling, and sharp pain) than other non-specific components (Lee et al., 2014). The posterior insular (pINS) and anterior cingulate cortices (ACC), which process ascending somatosensory and pain signals, showed acupuncture needling-specific brain responses in our previous study (Makary et al., 2018). Conversely, needling credibility has been shown to induce parasympathetic activation (decreased heart rate and pupil size responses), as well as vicarious acupuncture (*deqi*) sensations (deep pressure, heaviness, fullness, and numbness) (Lee et al., 2014). These vicarious sensations have been linked to increased brain response in the primary and secondary somatosensory cortices (S1 and S2) (Kerr et al., 2011; Beissner et al., 2015; Makary et al., 2018), and the evoked sensations may result from a top-down mechanism related to sensory imagery (Beissner et al., 2015) and attention toward stimulated body parts (Kerr et al., 2011). The previous study also showed that vicarious brain response (e.g., S1) and acupuncture sensations play an important role in creating and enhancing needling credibility in PHNT (Makary et al., 2018). Patients’ needling credibility was developed by the instruction that they would receive REAL stimulation and by the visual feedback being stimulated. Vicarious acupuncture (*deqi*) sensations induced by the visual feedback of acupuncture stimulation, mediated by the involvement of expectation (Song et al., 2019), mirror neuron system, or mirror-touch synesthesia (Beissner et al., 2015), seemed to bolster the needling expectancy and credibility.

Functional connectivity analysis has been used to investigate interactions between brain regions to better understand chronic pain mechanisms. Various clinical outcomes have been reported to be correlated with the functional connectivity within or between subregions in the default mode network (DMN), sensorimotor network (SMN), and salience network (SN). The strength of functional connectivity (between the DMN and SMN, as well as within DMN subregions) has been shown to be significantly correlated with the reported pain intensity in patients with chronic back pain (Hemington et al., 2018). In patients with chronic pelvic pain, functional connectivity between the anterior insular cortex (aINS; a subregion of the SN) and medial prefrontal cortex (mPFC; a subregion of the DMN) was positively correlated with the levels of anxiety, depression, and pain (As-Sanie et al., 2016). In patients with fibromyalgia, decreased DMN–insula connectivity was significantly positively correlated with decreased pain after acupuncture stimulation (Napadow et al., 2012). Functional connectivity between the insula and the somatotopic region of the leg in the S1 has been correlated with pain sensitivity in healthy controls (Kim et al., 2013) and with pain intensity in fibromyalgia patients (Kim et al., 2015).

Our previous study mainly focused on the brain responses to REAL and PHNT, as well as their neural correlates with clinical LBP levels (Makary et al., 2018). In this functional MRI (fMRI) study, however, the low back extension (LBE) pain model was used to evoke normalized back pain levels across patients, and functional connectivity analysis was performed to investigate the brain mechanism of pain modulation after REAL and PHNT. This allowed us to dissociate acupuncture-needling specific and non-specific components experimentally, as well as to understand the functional brain mechanisms of the short-term pain modulation in different brain hubs. In other words, the REAL was designed to encompass not only all of physical components of acupuncture stimulation (e.g., palpation, needle insertion, manipulation technique) as well as psychological components (e.g., needling credibility), while the PHNT was designed as a control in that this only has psychological components without any physical components. Thus, the aim of this study was to investigate the association between the physical components of acupuncture (controlled by the influence of psychological components) and brain functional connectivity.

## Materials and Methods

This study protocol was approved by the Institutional Review Board of the Kyung Hee University (KHNMC-OH-IRB 2010-013), and all participants provided written informed consent in accordance with the Declaration of Helsinki. This study is registered at the clinical research information service (CRIS)^1^ (registration number: KCT0002253).

### Participants

Fifty-six patients with non-specific low back pain (LBP; 31 men, age = 38.4 ± 12.7 years old, mean ± SD) were enrolled in this study. All patients completed prescreening for MRI eligibility and were included if they reported LBP of greater than four on a visual analog scale (VAS; 0 = no pain, 10 = most pain imaginable) when their low back was lifted (4–7 cm) in the supine position (for more on the LBE pain model, see below). Patients were excluded if they met the following exclusion criteria: (1) LBP greater than four during baseline, (2) severe pain other than LBP (e.g., neck pain) at scan, (3) severe radicular pain extending into lower leg, (4) psychiatric or cardiovascular disorders, (5) accident- or surgery-related back pain, (6) back pain from metastatic cancer, vertebral fracture, spinal infection, inflammatory spondylitis etc., (7) taking medication for pain management (e.g., corticosteroid, narcotics, muscle relaxants, and any herbal medicine), (8) receiving acupuncture treatment for back or neck pain within a month, and (9) participating any previous acupuncture studies.

### Experimental Paradigm

**FIGURE 1:**
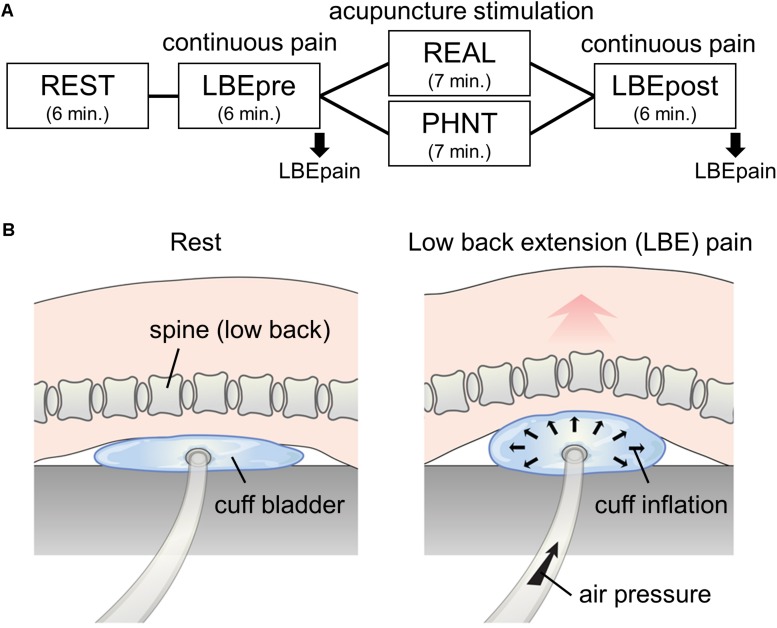
Experimental paradigm. **(A)** fMRI scanning protocol: 6-min resting state (REST) and continuous pain (LBEpre and LBEpost) runs with real (REAL) and phantom (PHNT) acupuncture stimulation. Pain model-induced low back pain (LBEpain) was recorded at the end of the scan. **(B)** Low back extension (LBE) pain model used in continuous pain runs.

The 56 enrolled non-specific LBP patients were randomized into either the REAL (*n* = 33) or PHNT (*n* = 23) group. Patients in both groups completed four fMRI runs: a resting-state run (REST, 6 min), an acupuncture stimulation run (REAL or SHAM, 7 min), and two continuous pain runs using the LBE pain model (6 min before and after the acupuncture stimulation run; these were named the LBEpre and LBEpost runs; Figure 1A). The perceived pain ratings throughout the LBE runs (LBEpain) were collected and its change (i.e., LBEpost – LBEpre) was analyzed as the main outcome measure in this study (Figure 1A).

All the setup equipment needed for the REAL and PHNT runs (MR-compatible acupuncture needles, camera, beam projector, screen, visual barrier, and blanket) was prepared before the MRI session (Makary et al., 2018). An MR-compatible cuff bladder was also placed on the MRI scanner bed for the two LBE runs (LBEpre and LBEpost), under the most painful area of the patients’ low back.

Before the LBEpre run, the cuff pressure for stimulation was calibrated in each individual patient. Air pressure was slowly applied to the cuff bladder until patients reported the target pain rating (4/10 on a 11-point VAS; 0 = no pain, 10 = most pain imaginable). In this way, we tried to standardize the intensity of induced LBP across all patients (Figure 1B). During this procedure, all patients confirmed that the exacerbated pain was due to their change in posture (extended back angle) rather than the force of the pressure. This information was recorded to ensure that the induced pain was not influenced by differing pain thresholds stimulating different layers of the body (e.g., skin, soft tissue, dura, disk, and deep muscle). During the LBEpre run, patients were asked to stay still with their eyes open, staring at crosshairs on a screen while the individually calibrated pressure was applied. They were also instructed to focus on the perceived LBP intensity and sensations. After the run, to prevent additional pain, the air pressure was cautiously removed so that the cuff bladder deflated slowly.

During the setup procedure for the REAL and PHNT runs, a video recording the patient body was projected onto the screen in real time to consolidate the video-body link between the patient’s own body and the displayed video. This visual feedback process was designed to initiate/boost needling credibility. Patients randomized into the REAL group were told that they would be stimulated with acupuncture needles (i.e., instruction) and received four real acupuncture needles (diameter = 0.3 mm, length = 30 mm; DongBang Co., Seongnam, South Korea): bilateral SP13, left SP11, and left ST36. Before the REAL run started, the needles were inserted at acupoints (which were chosen by a licensed and experienced acupuncturist based on their clinical effectiveness and easy access during the fMRI scanning) for clinical relevance and manipulated using the traditional technique by an acupuncturist to induce acupuncture (*deqi*) sensations. During the REAL run, the acupuncturist, who were trained for the stimulation paradigm, stimulated each needle in a random order [i.e., somatosensory needling afference; depth = 2–3 cm, 2 s per stimulation at 1 Hz rotation (±180°), five stimulations per acupoint, inter-stimulation interval = 7.9 ± 1.7 s], and the procedure was video-recorded and simultaneously played on the screen (i.e., visual feedback).

Before the PHNT run started, patients in PHNT group were also told that they would be stimulated with acupuncture needles (i.e., the same instruction given to REAL group) and after the video-body link was built by visual feedback procedure, the acupuncturist mimicked the needling ritual (i.e., insertion and manipulation) without actual needles. Patients in PHNT group were told that the needles had been inserted, as in the REAL run. The acupuncturist then pretended to stimulate the needles according to the stimulation paradigm while the previously recorded video from the other patient (in the REAL group, with real needles) was displayed on the screen during the PHNT run (i.e., visual feedback). Thus, the procedure was designed to build needling credibility based on the previous video-body link (during setup) and visual feedback (during the run). Importantly, this was carried out without any somatosensory needling afference.

For the LBEpost run, patients were stimulated with the same air pressure intensity with that was used in the LBEpre run. Subjective pain ratings (LBEpain, 0-10 VAS), as perceived throughout the LBE runs, were reported at the end of each scan, and the changes in LBEpain between LBEpost and LBEpre were calculated as the primary clinical outcome for pain reduction in REAL and PHNT (Figure 1A).

At the end of the experiment, patients’ needling credibility during REAL or PHNT runs was assessed retrospectively through an in-depth interview whether they believed that they had experienced REAL stimulation during the acupuncture stimulation run. If patients reported that they had any doubt or strong belief during the run that the stimulation was not happened to them actually (for example, due to no needling sensations), they were classified as non-credible patient group (i.e., without needling credibility) and removed from the analysis. Thus, our study simply hypothesized that REAL has somatosensory tactile stimulation, visual feedback, and needling credibility, whereas PHNT has only visual feedback and needling credibility.

### MRI Data Acquisition

MRI data were collected using a 3T Philips Achieva MRI scanner (Philips Medical Systems, Netherlands) with an 8-channel head coil at the Kyung Hee University Hospital, Gangdong. For anatomical localization of the results, structural T1-weighted images were collected using an MP-RAGE pulse sequence [repetition time (TR) = 9886 ms, echo time (TE) = 4.59 ms, flip angle = 8°, field of view (FOV) = 256 × 256 mm, voxel size = 1 × 1 × 1 mm], and functional images were acquired for 6 min using a T2^∗^-weighted echo planar imaging pulse sequence (TR = 2000 ms, TE = 35 ms, flip angle = 90°, FOV = 230 × 230 mm, voxel size = 2.875 × 2.875 × 4 mm, 34 interleaved axial slices) during the REST and LBE runs (LBEpre and LBEpost) for functional connectivity analysis. During fMRI, physiological data (electrocardiogram and respiration signal) were collected using a data acquisition system (PowerLab ML800; ADInstruments, Inc., Australia) to calibrate cardiorespiratory artifacts (Glover et al., 2000).

### Data Processing and Analysis

Collected MRI data were processed using conventional analysis software, such as the Functional Magnetic Resonance Imaging of the Brain (FMRIB), Software Library (FSL)^2^, Analysis of Functional NeuroImages (AFNI)^3^, and FreeSurfer^4^. The fMRI data were corrected for cardiorespiratory artifacts (3dretroicor, AFNI) (Glover et al., 2000), and for head motion (MCFLIRT, FSL; ICA-AROMA). They were also preprocessed for skull stripping (BET, FSL), spatial smoothing (Gaussian kernel, full width at half maximum = 6 mm; fslmaths, FSL) and temporal filtering (high-pass cut-off frequency = 0.006 Hz; 3dBandpass, AFNI). Individual structural and functional data were aligned first (bbregister, FreeSurfer). They were then co-registered to the standard Montreal Neurological Institute (MNI) space (FNIRT, FSL) to allow dual-regression independent component analysis (ICA) (Filippini et al., 2009) and seed-voxel connectivity analysis.

In the dual-regression ICA, all fMRI data from the REST, LBEpre, and LBEpost runs were temporally concatenated and then fed into the group ICA (MELODIC, FSL). The group ICs of the DMN, SMN, and SN were selected based on the spatial templates of the resting state networks (Beckmann et al., 2005). ROIs in the DMN (posterior cingulate cortex, PCC; mPFC) and SN (anterior and middle cingulate cortices, aINS and pINS) were decided based on the group map of REST run to ensure they were independent of influence from the pain model and acupuncture stimulation. ROIs in the aINS and pINS were combined bilaterally. The ROI in the SMN was located in the bilateral low back region of the primary somatosensory cortex (S1_back_; MNI *X* = ± 18 mm, *Y* = −38 mm, *Z* = 72 mm) (Table 1) based on a localization run in an independent LBP study (Lee et al., 2018a) that investigated low back-specific functional connectivity. The average time-series brain activities (sphere mask, radius = 4 mm) were extracted from each of the ROIs, and the correlation coefficients (Pearson’s *r*) of cross-correlation between the ROIs were converted into *z* scores using Fisher *z*-transformation. The changes in within- and between-network connectivity (LBEpost vs. LBEpre) after REAL and PHNT runs, as well as their association with changes in LBEpain (primary clinical outcome), were calculated (Hemington et al., 2018). Significance was defined at *P-*values <0.05.

**TABLE 1:** Locations of regions of interest in functional connectivity analysis.

**Network**	**ROI**	**Side**	**MNI coordinates (mm)**		
			**X**	**Y**	**Z**
DMN	mPFC	R	2	64	−8
	PCC	L	−6	−40	24
SMN	S1_back_	R/L	±18	−38	72
	dACC/aMCC	R	4	24	26
SN	aINS	R	38	10	4
		L	−36	12	2
	pINS	R	46	−4	2
		L	−42	−16	−2

*ROI, region of interest; MNI, Montreal Neurological Institute; DMN, default mode network; SMN, sensorimotor network; SN, salience network; mPFC, medial prefrontal cortex; PCC, posterior cingulate cortex; S1_*back*_, low back region in primary somatosensory cortex; dACC, dorsal anterior cingulate cortex; aMCC, anterior middle cingulate cortex; aINS, anterior insular cortex; pINS, posterior insular cortex; R, right hemisphere; L, left hemisphere.*

## Results

**FIGURE 2:**
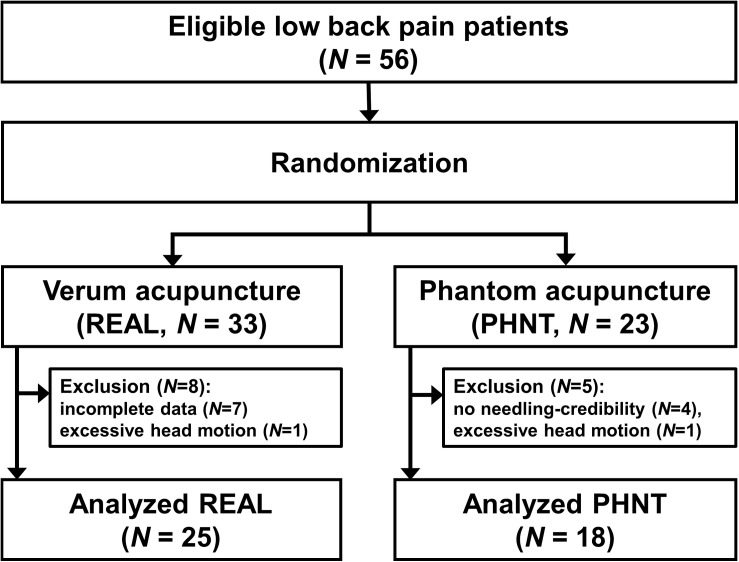
Flow chart for enrollment, randomization, exclusion, and analysis.

Of the 56 enrolled LBP patients (33 in the REAL group and 23 in the PHNT group), 43 were included in this analysis (25 in the REAL group, 38.4 ± 13.2 years old, mean ± SD, 13 men; 18 in the PHNT group, 38.3 ± 13.0 years old, 8 men; *P* for age [REAL vs. PHNT] = 0.98). Eight patients were excluded from the REAL group: four due to incomplete scanning, three due to uncorrectable distortions, and one due to excessive head motion (>2 mm). Five were excluded from the PHNT group: four due to the absence of needling credibility (patients reported that the acupuncture procedure did not happen to them, and no acupuncture stimulation was delivered because they did not feel any sensation during the PHNT run) and one due to excessive head motion (>2 mm) (Figure 2).

### Decreased Pain Model-Induced Pain Intensity (LBEpain) After the REAL and PHNT Stimulation

While the applied air pressure for pain model was individually calibrated and applied with the same intensity in LBEpre and LBEpost [REAL: 118.4 ± 72.4 mmHg, PHNT: 94.4 ± 57.2 mmHg; *P* (REAL vs. PHNT) = 0.25], the PHNT group reported significantly greater LBEpain than the REAL group during the LBEpre run [REAL: 4.74 ± 1.00/10, PHNT: 5.54 ± 0.83; *P* (REAL vs. PHNT) = 0.01], and the reported pain was significantly greater than the target value (4/10) in both groups [*P* (REAL) < 0.005, *P* (PHNT) < 0.001]. None of the patients reported any pain during the REST run.

**FIGURE 3:**
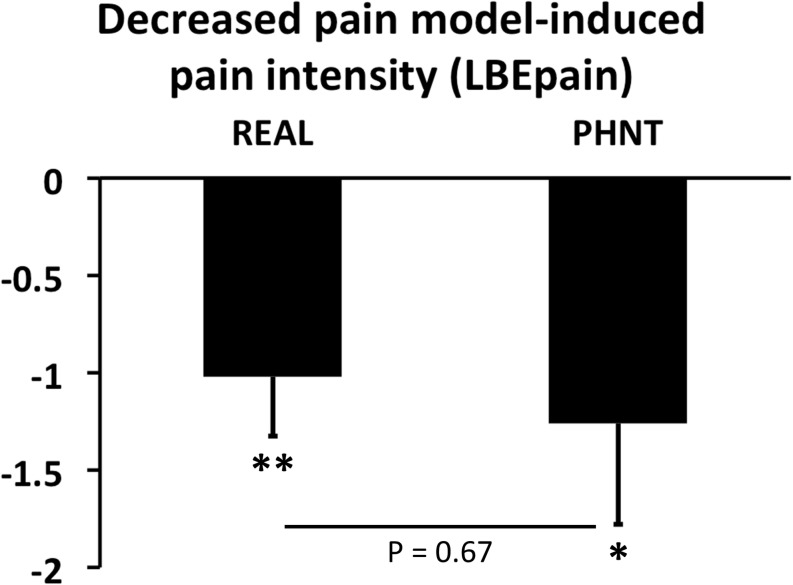
Changes in LBEpain during continuous pain runs. Real (REAL) and phantom (PHNT) acupuncture stimulation showed significant short-term pain-relieving effects (*P* < 0.01 and 0.05, respectively) without any difference between groups (*P* = 0.67). N.B., bar plots show mean ± SEM. ^∗^*P* < 0.05; ^∗∗^*P* < 0.01.

Importantly, however, the subjective pain (LBEpain) which patients felt during the continuous pain runs, as measured using the VAS, was significantly reduced after both REAL and PHNT runs [Δ = LBEpain(LBEpost) – LBEpain(LBEpre); ΔREAL: −1.02 ± 1.53, *P* < 0.005; ΔPHNT: −1.26 ± 2.20, *P* < 0.01; *P* (ΔREAL vs. ΔPHNT) = 0.67] (Figure 3).

In spite of the randomization of the groups, the LBEpain in LBEpre was significantly different in the REAL and PHNT. Thus, for the analysis of brain correlates, we investigated how the LBEpain reduction was correlated with the change of functional connectivity (LBEpost vs. LBEpre).

### Changes in Functional Connectivity and Its Association With Clinical Outcome

Twenty-five ICs were derived from the dual-regression group ICA, and the ICs in the DMN, SN, and SMN were identified. The ROIs were then decided in the submodules of each network (Table 1 and Supplementary Figure S1). The DMN mainly comprised the PCC, inferior parietal lobule, and the mPFC. The ROIs in the PCC and mPFC were localized in DMN. The SMN encompassed the pre- and post-central gyri, including the predefined S1_back_ ROI. The SN consisted of the dorsal anterior and anterior middle cingulate cortices, as well as the supplementary motor area, ventrolateral prefrontal cortex, and the aINS and pINS on both sides. The pINS is the main subregion of the SMN, as reported in a previous study involving 1000 healthy controls (Yeo et al., 2011). However, in the present study involving patients with LBP, a substantial portion of the pINS was intrinsically connected with the SN (Supplementary Figure S1).

**FIGURE 4:**
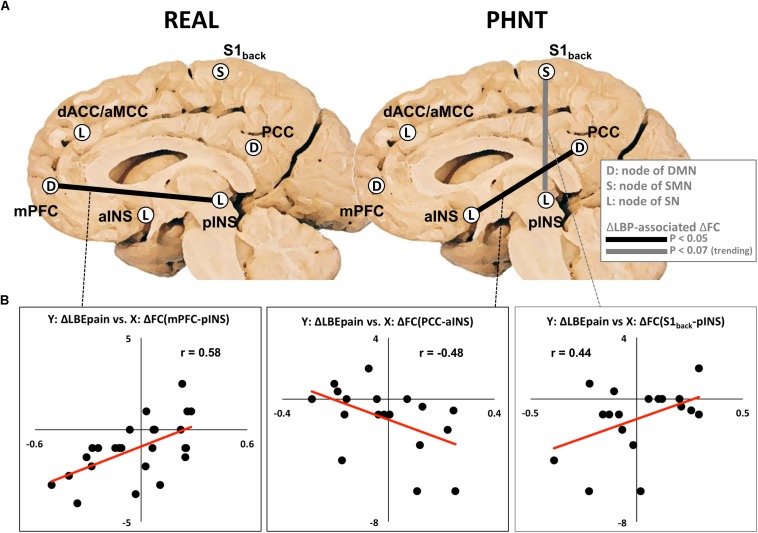
Relationship between changes in functional connectivity and changes in LBEpain (LBEpost vs. LBEpre) after real (REAL) and phantom (PHNT) acupuncture stimulation. **(A)** Functional connectivity changes (mPFC–pINS in REAL, PCC–aINS, and S1_back_–pINS in PHNT) associated with decreased LBEpain. Increased functional connectivities (dACC–MCC and aINS–pINS) were found in PHNT. **(B)** Correlation between changes in LBEpain (*Y*-axis) and functional connectivity (*X*-axis). DMN, default mode network; SMN, sensorimotor network; SN, salience network; FC, functional connectivity; LBP, low back pain; mPFC, medial prefrontal cortex; PCC, posterior cingulate cortex; S1_back_, low back region in primary somatosensory cortex; dACC, dorsal anterior cingulate cortex; aMCC, anterior middle cingulate cortex; aINS, anterior insular cortex; pINS, posterior insular cortex; *Y*, *Y*-axis; *X*, *X*-axis.

While no significant differences in functional connectivity were found between the LBEpre and LBEpost runs, we found that the REAL group showed significantly positive correlation (*r* = 0.58, *P* < 0.01) between the reduction in LBEpain and the change of functional connectivity between the nodes of the DMN (mPFC) and SN (pINS) (Figure 4).

In the PHNT group, the change in LBEpain was negatively correlated with the change of DMN–SN connectivity (PCC–aINS, *r* = −0.48, *P* < 0.05), and it showed a trending positive correlation with the change of SMN–SN functional connectivity (S1_back_-pINS, *r* = 0.44, *P* = 0.07) (Figure 4).

Interestingly, there was no significant difference between the REAL and PHNT groups in terms of changes in functional brain connectivity (LBEpost – LBEpre) as well as any other significant correlations between the reduction in LBEpain and the change of functional connectivity between the nodes of the DMN, SN, and SMN.

## Discussion

In the present study, we investigated the differential influence of needling-specific and non-specific components in acupuncture treatment using REAL and PHNT, whereby REAL induced needling credibility by combining somatosensory needling afference and visual feedback from the video, while PHNT did so through visual feedback only. The LBE pain model was used to modulate and standardize the patients’ back pain levels, while functional connectivity analysis was performed to investigate the brain mechanism of the short-term pain modulation effects of REAL and PHNT. We found that the LBE pain model significantly increased clinical pain levels (LBEpain) in patients with back pain, and that this elevated LBEpain was reduced after both REAL and PHNT stimuli, indicating that both methods shared a common, short-term, pain-relieving effect. However, the corresponding brain mechanisms for pain reduction differed from each other. Somatosensory afference seemed to play an important role in pain modulation in REAL, because the physical-pain processing area (pINS) was engaged during corresponding functional connectivity alteration. In PHNT, a cognitive/affective factor (needling credibility and anticipation) seemed to be involved in the relevant connectivity changes, namely the salience-processing area (aINS). These results allowed us to identify short-term brain connectivity changes related to acupuncture-specific or non-specific effects on back pain intensity (LBEpain).

### Clinical Pain Reduction in REAL: Detachment of Self From Pain

In REAL, greater LBEpain reduction was associated with decreased connectivity between the mPFC and pINS. Meta-analysis of acupuncture stimulation has shown significant deactivation in the mPFC and PCC, indicating that these areas modulate pain by shifting the focus between internal physical/mental states and external stimulation (Chae et al., 2013). Deactivation in the DMN subregion has also been observed both during real and sham acupuncture stimulation (Jung et al., 2015; Makary et al., 2018; Zhang et al., 2019). Previous studies have shown that the mPFC plays an important role in self-referential processing, which is critical for physical signal regulation, including pain (Stankewitz et al., 2018). Such processing includes both positive and negative self-appraisal (Dixon et al., 2017), as well as self-rumination and pain catastrophizing (Lee et al., 2018b). This consistent deactivation in the mPFC was interpreted as an attentional shift from self-referential to external-focused attention for a better understanding of given events and stimulations. The pINS shows stimulation-specific responses to the physical aspects of pain, representing bottom-up processing of sensory information. Thus, activity in the pINS has been considered a proxy of somatosensory (Christopher et al., 2014), pain (Morel et al., 2013), pain ratings (Segerdahl et al., 2015), and cortical amplification (Lee et al., 2017) processing. It is likely for this reason that activation in the pINS has been reported in most acupuncture neuroimaging studies (Chae et al., 2013). Indeed, it has recently been closely linked to the needling-specific component of acupuncture (somatosensory afference and corresponding arousal) (Jung et al., 2015; Makary et al., 2018). Interestingly, the pINS has been reported to be the main subregion of the SMN in healthy controls (Yeo et al., 2011), because it is significantly engaged in the physical aspects of pain and sensory processing. However, in the present study, a significant portion of the pINS was a part of the SN, rather than the SMN. The physical aspect of back pain, including painful sensations, seemed to capture the patients’ attention, at least during the scan runs, in contrast to the healthy controls. This speculation should be further investigated.

Interestingly, the association between DMN and INS functional connectivity and clinical pain levels has been reported in other studies. For example, in patients with fibromyalgia, reduced clinical pain has been associated with reduced DMN–INS connectivity, and the same connectivity has been suggested as a possible marker for chronic pain (Napadow et al., 2012). In patients with low back pain, DMN–pINS connectivity, as well as changes thereof, has been shown to be significantly correlated with baseline (and increased) clinical pain (Loggia et al., 2013). The same relationship has also been reported in patients with LBP, complex regional pain syndrome, and knee osteoarthritis (Stankewitz et al., 2018). Thus, it has been speculated that a greater awareness of pain and the stronger incorporation of pain within the self (increased DMN–INS connectivity) can predict higher pain ratings (Napadow et al., 2012; Loggia et al., 2013). In other words, patients detach themselves from the pain, they report lower levels.

Our findings in REAL are in line with previous studies. Thus, we speculated that detachment of self from the physical aspects of pain contributes to the reduction in back pain (LBEpain) after REAL. As somatosensory tactile input is a predominant factor in creating sensory perception (e.g., reported strong acupuncture sensations), we speculated that the influence of somatosensory afference may play a crucial role in the relationship between changes in functional connectivity and low back pain.

### Clinical Pain Reduction in PHNT: Endorsement of Self and Disengagement in Pain Processing

In PHNT, we found that the increase in functional connectivity between PCC and aINS was associated with reduced LBEpain. The PCC is the main node of the DMN (Zhang et al., 2019), which is associated with self-reflection (Vogt and Laureys, 2005), internally directed cognition (Leech and Sharp, 2014), bodily attention (Jung et al., 2015), and self-referential pain catastrophizing (Lee et al., 2018b). In many acupuncture studies, consistent deactivation in the PCC has been reported in both real and sham acupuncture stimulation (Huang et al., 2012; Chae et al., 2013; Makary et al., 2018). The aINS, as part of the SN, is a key region in directing cognitive process (Christopher et al., 2014), transient attention (Cauda et al., 2012), significance of stimulus (Christopher et al., 2014), and empathy with perceptive-taking (Nieuwenhuys, 2012; Christopher et al., 2014), as well as in the integration of anticipation and sensory inputs (Jung et al., 2015), cognitive and affective processing of pain (Morel et al., 2013; Kuehn et al., 2016; Stankewitz et al., 2018), and self-awareness of physical condition (Stankewitz et al., 2018). In our previous study, increased activity in the aINS and decreased activity in the PCC were observed during both REAL and PHNT, and aINS activity was positively correlated with changes in low back pain (Makary et al., 2018). Thus, neither region is specific to the physical aspects of stimulation, but rather to the cognitive/emotional aspects of it – the PCC to self-referential processing and endorsement of self, and the aINS to processing of the salient/emotional aspects of pain events.

**FIGURE 5:**
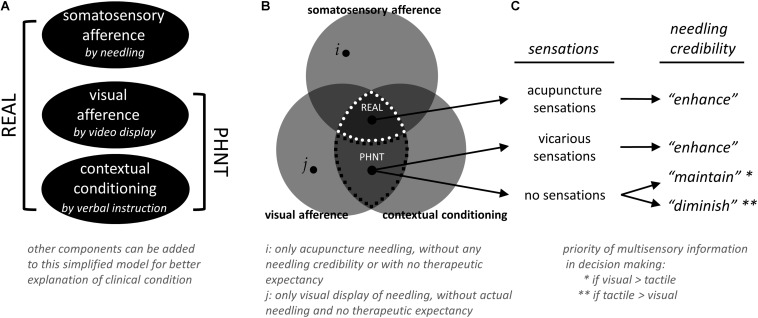
Simplified model for real (REAL) and phantom (PHNT) acupuncture, and needling credibility. **(A)** Different combinations of components have been defined and used to model REAL and PHNT conditions and to explain the influence of each component experimentally. For better explanation and understanding of clinical condition, other components can be added to this simplified model. **(B)** Other than REAL and PHNT conditions (defined by somatosensory afference, visual afference, and contextual conditioning), several other tests can be done with different combination of components. For example, conditions with only acupuncture needling (*i*, without any needling credibility/therapeutic expectancy and visual display of needling) and with only visual display of needling (*j*, without actual needling and needling credibility/therapeutic expectancy) can be tested. Rest of those regions were not tested in this study (based on our experience, visual afference and contextual conditioning themselves might be able to “*initiate*” the needling credibility, but it was not explicitly tested in this study). **(C)** Needling credibility was influenced by a relationship between visual and tactile afferences from video and needling, respectively, contextual conditioning from treatment instruction, and belief of treatment from the in-depth interview with subjects.

We observed a different pattern during PHNT than during REAL. Specifically, the aINS, which is the center of cognitive/emotional pain processing, as well as salience, plays an important role in pain reduction during PHNT, whereas the pINS, which is the center for physical pain processing does so during REAL. In PHNT, needling credibility can be consolidated by visual information and feedback from acupuncture stimulation, without somatosensory tactile inputs. This needling credibility might be built as a result of an internal decision-making process against incongruent visual and tactile information (i.e., between visual information which shows stimulation ritual and no/less sensations felt in their body) (see section “Empirical Account for Needling Credibility,” Figure 5). In addition, vicarious sensations might be a result of shift the focus (the aINS) from the physical aspects of pain to the self (the PCC) as they believed they were actually stimulated with needles. Thus, this attentional shift from external pain to the self (increased PCC–aINS connectivity) might be related to LBEpain reduction in PHNT. We speculated that engagement of emotional regulation and subsequent disengagement between pain processing regions may contribute to the improvement in symptoms when there is a cognitive/affective component induced by needling credibility, not by needling (placebo effect).

Interestingly, the decreased level of functional connectivity between the S1_back_ and pINS, which was transited from SMN to SN, tended toward correlation with the reduction in LBEpain (*P* = 0.07) in PHNT. A functional connection between the pINS and S1 has been reported, and it may result in precise perception and awareness of pain (Christopher et al., 2014). In one study involving healthy controls, S1–pINS connectivity that was somatotopically specific to the leg was negatively correlated with pain sensitivity when cuff stimuli were applied to the leg (Kim et al., 2013), and the S1 has shown significantly stronger pain-specific functional connectivity to the pINS (Peltz et al., 2011). S1–aINS connectivity has been reported to be significantly correlated with clinical pain in fibromyalgia, suggesting that synchronization between pain-processing regions influences exacerbated pain states (Kim et al., 2015). Thus, our results might suggest that bottom-up nociceptive sensory information is delivered to the corresponding S1 subregion (S1_back_), as well as to the physical pain-processing area (pINS), and desynchronization between these two regions may ameliorate back pain (LBEpain) in PHNT. Further study, however, should be followed to investigate this speculation.

### Empirical Account for Needling Credibility

Our experimental paradigm was designed to build and enhance needling credibility by an interaction between somatosensory needling afference from needling, visual feedback from the video, and instruction from the acupuncturist. Different combinations of components have been defined and used to model REAL and PHNT conditions and to explain the influence of each component experimentally (Figure 5A). In the first PHNT acupuncture experiment (Lee et al., 2014), 20 healthy participants received both REAL and PHNT stimulation in a counterbalanced design. After an in-depth interview with all subjects, eleven subjects of 20 were found to have needling credibility during the experiment and nine were not. Interestingly, some of the subjects believed they were actually stimulated with REAL during PHNT stimulation; some reported retrospectively that they thought the acupuncture stimulation was applied with new and advanced techniques so that they didn’t feel much sensations, and some actually reported vicarious sensations. This phenomenon–reporting sensations in observing other’s stimulation–has been reported and discussed as results of expectation (Song et al., 2019), sensory mirror system or mirror-touch synesthesia (Beissner et al., 2015; Makary et al., 2018), or body-ownership (Botvinick and Cohen, 1998). This implies that instruction to the subjects and visual feedback are also important factors to create those sensations, where the contribution of needling credibility to vicarious sensation is not significant (i.e., not all subjects with needling credibility reported vicarious sensations). However, four subjects who didn’t believe that they were stimulated emphasized synchronization problem between the visual feedback from the video and the actual movement of an acupuncturist. Other five answered that they did not feel any sensation at the stimulation site or surrounding body region during PHNT stimulation, thus they thought the stimulation was not actually applied to them. In the second study (Makary et al., 2018), four LBP patients were excluded from the analysis as they reported the absence of needling credibility during the PHNT run. The major reason was no acupuncture sensations during the stimulation period, too. This shows that the existence of acupuncture sensations is an important factor for needling credibility. Rest of the patients, who believed that they were actually stimulated with needles, reported vicarious sensations or did believe even without any sensations from the stimulation. Thus, once the sensations (either actual or vicarious) were perceived, subjects tend to believe that they were actually stimulated with needles. If there were no sensations felt from the stimulation (while the instruction was given for needling), their needling credibility seemed to rely on the individually different weighting/priority of multisensory information in decision making (i.e., shift attention to self). This gives clue to estimate who will be likely to have needling credibility and who would not in the PHNT stimulation paradigm.

Taken together, (1) instructions from acupuncturist that they would be stimulated with real acupuncture needles and (2) visual feedback that is a simultaneous display of acupuncture stimulation (i.e., observation of treatment procedure with REAL) contributes to induce vicarious sensations and to *initiate* needling credibility (even without actual needling). (3) Actual or vicarious acupuncture sensations play an important role in *enhancing* needling credibility. (4) If tactile/acupuncture sensations are not congruent with visual feedback (for example, when they are observing the treatment procedure but do not feel any sensation from it), individual priority of multisensory information might affect maintaining the needling credibility or diminishing/eliminating of it (Figures 5B,C). Further studies should be done to confirm this hypothesis with several experimental conditions: (1) for better explanation and understanding of clinical condition, other components can be added to our model (Figure 5A), (2) conditions with only acupuncture needling (without any needling credibility/therapeutic expectancy and visual display of needling) and with only visual display of needling (without actual needling and needling credibility/therapeutic expectancy) (Figure 5B, i and j, respectively), (3) with controlling the influence of instructions (Cheon et al., 2018), (4) with different contents of provided visual information (i.e., treatment-relevant or not-relevant, or incongruent with actual stimulation), and (5) with different extents to which patients pay attention to the given visual or perceived sensations.

### Limitations

Several limitations in the present study must be noted. Firstly, we had no untreated control group in which to investigate the effect of elapsed time (e.g., habituation or sensitization to our pain model), nor did we include a healthy control group to identify patient-specific brain responses (e.g., the involvement of the pINS in SN and connectivity changes to the pain model). Secondly, the number of subjects who experienced no needling credibility was too small (*n* = 4) to dissociate the effect of needing credibility from the effect of visual feedback in PHNT. As most of the patients who received PHNT believed that they had been treated with REAL and reported vicarious acupuncture (*deqi*) sensations, a separate group with enough number of the subject must be included in future studies to explicitly exclude needling credibility during REAL/PHNT session to investigate the influence of needling credibility. Thus, several experimental conditions (with and without somatosensory needling, needling credibility, instruction, and visual feedback etc.) with a sufficient number of the subject are needed to investigate the analgesic effects of somatosensory and cognitive/affective components on back pain. For example, shallow (minimal acupuncture or tactile stimulation) acupuncture to investigate the influence of the amount of somatosensory afference, acupuncture methodology (manual- vs. electrical-acupuncture stimulation), acupoint specificity (by comparing sham points and acupoints), and no/irrelevant display to patients to inquire into the influence of the visual feedback can be used. Thirdly, along with the limitation of experimental conditions, several cognitive/affective factors were not considered in this analysis. Thus, for better understanding future study should also investigate factors which are clinically relevant and influential: doctor–patient relationship (rapport), previous acupuncture experience and its efficacy, expectancy of acupuncture efficacy, accuracy and consistency of sensation reporting, degree of sleepiness/awakeness/engagement during the experiment, and eagerness to be cured. Specific non-needling factors (i.e., acupuncture theory-based factors such as diagnosis and palpation) were not considered in this study. Fourthly, the acupoints were decided for the general purpose of back pain reduction and were not individualized to maximize its efficacy. Further study needs to be done with a set of individualized acupoints to further understand the influence of different aspects of acupuncture. Lastly, randomization should have performed more accurately and systematically. Unbalanced number of patients were allocated for REAL (*n* = 33) and PHNT (*n* = 23). Also, the significantly different baseline (LBEpre) pain scores made it difficult to investigate its direct comparison between groups, and thus the analysis of this study was limited to the changes in pain scores.

### Conclusion

In the present study, we found significant back pain reduction and corresponding changes in functional connectivity after REAL and PHNT. It suggests that the involvement of different brain regions is related to improved symptoms, for example by detaching self (DMN) from the sensory aspect of pain (pINS) in REAL, by shifting attention (aINS) to self (DMN), and by disengaging between physical pain processing hubs (e.g., pINS and S1_back_ region) in PHNT. We also speculated the relationship between visual and tactile information from video and needling, respectively, contextual conditioning from treatment instruction, and belief of treatment. This experimental paradigm might provide an appropriate sham methodology to dissociate the influence of different acupuncture components in acupuncture study, and the findings might help to understand corresponding brain mechanisms of acupuncture analgesia.

## Ethics Statement

This study protocol was approved by the Institutional Review Board of the Kyung Hee University (KHNMC-OH-IRB 2010-013), and all participants provided written informed consent in accordance with the Declaration of Helsinki. This study is registered at the clinical research information service (CRIS; http://cris.nih.go.kr/) (registration number: KCT0002253).

## Author Contributions

JL, JK, and KP contributed to the conception and design of the study. JL, SE, J-HL, and KP conducted the experiment and collected the data. JL and SE performed the statistical analysis. JL, SE, and KP interpreted the results and wrote the manuscript. All authors contributed to the manuscript version, read, and approved the submitted version.

## Conflict of Interest

The authors declare that the research was conducted in the absence of any commercial or financial relationships that could be construed as a potential conflict of interest.

## Footnotes

1 http://cris.nih.go.kr/

2 https://fsl.fmrib.ox.ac.uk/fsl/fslwiki/

3 https://afni.nimh.nih.gov/

4 https://surfer.nmr.mgh.harvard.edu/fswiki
