# Supplementary material for: Differential Influence of Acupuncture Somatosensory and Cognitive/Affective Components on Functional Brain Connectivity and Pain Reduction During Low Back Pain State
Source: Front Neurosci. 2019 Oct 4;13:1062. doi: 10.3389/fnins.2019.01062 (PMC6788296; doi:10.3389/fnins.2019.01062)
Supplement: Supplementary file 1 [file Data_Sheet_1.PDF]

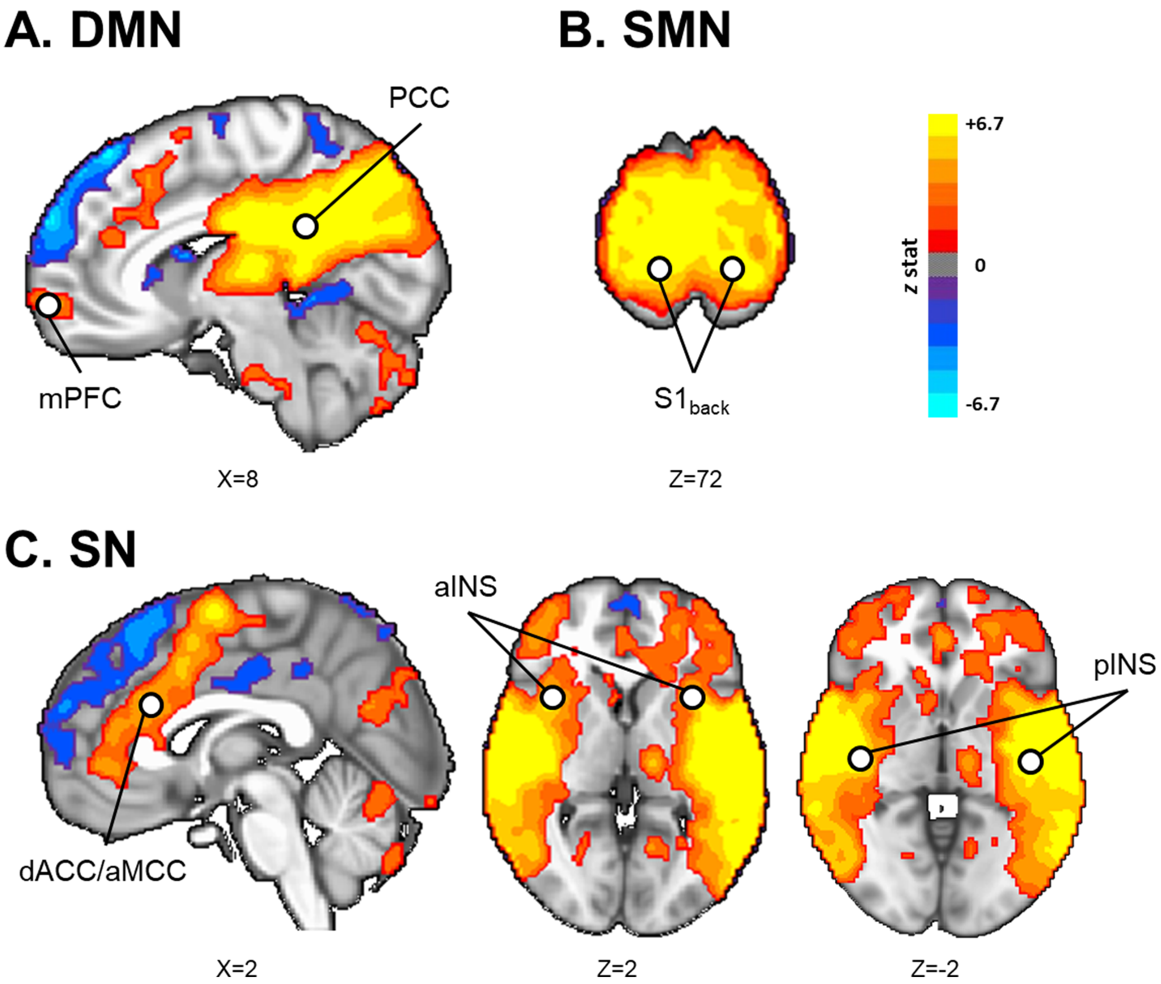

Supplementary Figure 1. Locations of regions of interest in (A) DMN, (B) SMN, and (C) SN for functional connectivity analysis. DMN, default mode network; SMN, sensorimotor network; SN, salience network; mPFC, medial prefrontal cortex; PCC, posterior cingulate cortex; $S1_{back}$, low back region in primary somatosensory cortex; dACC, dorsal anterior cingulate cortex; aMCC, anterior middle cingulate cortex; aINS, anterior insular cortex; pINS, posterior insular cortex; Y, Y-axis; X, X-axis.
